# Mpox stigma in the UK and implications for future outbreak control: a cross-sectional mixed methods study

**DOI:** 10.1186/s12916-025-04243-3

**Published:** 2025-07-15

**Authors:** Amy Paterson, Ashleigh Cheyne, Harun Tulunay, Chloe Orkin, Will Nutland, Jake Dunning, Jeni Stolow, Nina Gobat, Piero Olliaro, Amanda Rojek, Outbreak Stigma Assessment Expert Group

**Affiliations:** 1 Pandemic Sciences Institute, University of Oxford, Old Road Campus, Roosevelt Drive, Oxford, OX3 7DQ UK; 2 Positively UK, 14 St. Marys House, Chillingworth Road, London, N7 8SH UK; 3 Centre for Immunobiology, SHARE Collaborative, Blizard Institute, Queen Mary University of London, London, UK; 4 The Love Tank, The Green House, 244-254 Cambridge Heath Road, London, E2 9DA UK; 5 Tulane University School of Public Health and Tropical Medicine, New Orleans, LA USA; 6 Global Outbreak Alert and Response Network, World Health Organization, Geneva, Switzerland; 7 Nuffield Department of Primary Care Health Services, University of Oxford, 32 Woodstock Road, Oxford, OX2 6HT UK

**Keywords:** Discrimination, Emerging infectious disease outbreak, Health policy, Mpox, Prejudice, Public health, Stigma

## Abstract

**Background:**

Stigma emerged as a prominent public health challenge in the global mpox outbreak that began in 2022, impeding outbreak control efforts and the well-being of affected individuals. Addressing stigma is important for improving infection prevention and control. Despite frequent mention in public and policy discourse, robust assessment of mpox stigma is lacking. This study investigated the causes, manifestations, and impacts of mpox-related stigma in the UK, focusing on anticipated stigma among directly and indirectly affected communities.

**Methods:**

We conducted an online, mixed-methods cross-sectional survey to assess mpox stigma. We developed and content validated a new tool, the Stigma Survey and Community-based Assessment for New and Re-emerging outbreaks (Stigma-SCANR) for this purpose. Through quota sampling, the survey targeted populations most affected by mpox at the time of data collection (March–July 2024), including gay, bisexual, and other men who have sex with men (GBMSM), and healthcare workers. The survey primarily explored anticipated stigma. Respondents with previous mpox diagnoses were asked about personal experiences of stigma.

**Results:**

Of 479 respondents who initiated the survey, 437 (91%) were included in analyses. In modules related to drivers of stigma, pre-existing prejudices towards associated groups such as GBMSM and migrants were emphasised, alongside fear and misinformation. On average, respondents anticipated higher levels of negative judgement and unwarranted avoidance compared to other forms of social stigma, particularly from casual partners and the public. Among the 13 respondents who reported a previous mpox diagnosis, 11 (85%) had experienced mpox-related stigma. Nearly a quarter of respondents (24%) said they would not, or were unlikely to, tell a recent sexual partner about an mpox diagnosis. Feelings of shame were considered the most common barrier to care-seeking (299 respondents, 68%).

**Conclusions:**

This analysis of mpox stigma in the UK offers insights for international outbreak response, particularly in countries with similarly affected communities. Lessons learnt may also be transferable to other disease outbreaks. We propose practical recommendations for reducing stigma in future outbreaks, including peer support initiatives, distributing accessible information about safe timelines for returning to socialising and work or school, and co-designing public communications and contact tracing programmes with affected community members.

**Supplementary Information:**

The online version contains supplementary material available at 10.1186/s12916-025-04243-3.

## Background

Mpox has been a persistent health problem in affected regions of Africa for decades. However, since 2022 a growing number of cases have been reported outside of these countries [1]. This prompted the World Health Organization (WHO) to declare two successive Public Health Emergencies of International Concern, first due to the global spread of clade IIb mpox virus (MPXV) (2022–2023), and then due to a surge of clade I MPXV, including a new clade (Ib) (2024-ongoing) [1]. Mpox outbreak control, with an emphasis on effective community engagement and risk communication, is a global health priority [2].

The United Kingdom (UK) was among the first countries to detect MPXV clade IIb during the 2022 global outbreak and remains one of the most affected, with over 4000 confirmed cases to date (as of May 2025) [1, 3]. A distinctive feature of the clade IIb outbreak in newly affected countries was sustained transmission during sexual contact primarily within, but not restricted to, networks of gay, bisexual, and other men who have sex with men (GBMSM) [4].

Stigmatisation is understood as an interactive social process in which an individual or group is disqualified from full social acceptance due to an attribute (in this case an illness) their society considers discrediting [5, 6]. This umbrella term includes the endorsement (by others or oneself) of negative stereotypes, referred to as ‘prejudice’, and behavioural manifestations or differential treatment leading to disadvantage, referred to as ‘discrimination’ [7, 8].

Mpox stigma is known to contribute to mental health challenges, as well as hesitation regarding care-seeking, vaccination, and isolation [4, 9–11]. Although stigma is often mentioned in popular media and policy discussions, structured investigations into its specific forms, root causes, and effects on outbreak control are limited.

Addressing stigma is crucial for community well-being and effective public health responses to infectious disease outbreaks [2, 7]. Understanding its drivers and impact can inform stigma reduction within public health policies [12]. This study examined mpox stigma in the UK, with a particular focus on perceived and anticipated stigma, and its impact on outbreak control. The study proposes recommendations to mitigate stigma and tailor response efforts in future public health crises.

## Methods

We conducted a cross-sectional mixed methods study using an online survey. We co-developed and implemented a new public health tool, the Stigma Survey and Community-based Assessment for New and Re-emerging outbreaks (Stigma-SCANR), for this purpose with affected community members and outbreak response experts. We report our methods and findings according to the Consensus-Based Checklist for Reporting of Survey Studies (CROSS) guidelines (Additional file 1: Table S1) [13].

### Sampling and recruitment

The sampling frame for this study consisted of UK residents aged 18 years or older who are part of communities directly or indirectly affected by the mpox outbreak. The survey was open from March to July 2024.

We calculated a required total sample size of 355 respondents, using Epitools [14], with a maximum margin of error of ± 5% at 95% confidence, and 36% estimated prevalence of stigma based on a recent systematic review and meta-analysis of stigma in infectious disease outbreaks [15]. Under the conservative assumption *p* = 0.5 (which maximises variance to address the risk of misestimation), the required sample would increase to 385. We over-recruited by approximately 25% to account for ineligible or incomplete responses.

We employed a non-probabilistic purposive sampling approach, with quotas established for (a) community members considered at higher risk (predominantly GBMSM) (78% of sample) and (b) healthcare workers (including those who also identify as GBMSM) (22% of sample), as key groups eligible for the national mpox vaccination programme at the time [16]. Quotas were based on estimated population sizes for these population groups in the UK [17–20]. The quotas were used to inform recruitment but were not enforced. Similarly, since sampling was non-probabilistic, the sample size calculation served as a guide for recruitment logistics rather than a strict measure for ensuring generalisability.

We recruited respondents through the online survey platform Prolific, using filters for UK residents who identify as LGBTQ + men or as healthcare workers, applying the above quota proportions. We also recruited through social media and institutional mailing lists of local HIV and LGBTQ + organisations and sexual healthcare and outbreak response professional networks (HIV i-Base, the British HIV Association, the British Association for Sexual Health and HIV, and the International Severe Acute Respiratory and emerging Infection Consortium) as well as individual HIV and LGBTQ + advocates.

Following consultation with community contributors and field experts, we did not exclude individuals who engaged with these recruitment channels and were aware of mpox, but who did not identify as LGBTQ + men or healthcare workers in the survey. This decision aimed to maximise inclusivity, in recognition that the outbreak was not confined to these groups.

Respondents recruited through Prolific were reimbursed £2.50 each, in line with the platform’s recommended reimbursement rates. To preserve anonymity, those who responded directly through social media and mailing lists were not reimbursed, as this would require collecting personal details. This was clearly communicated in the information sheet.

### Data collection tool

#### Development and validity

The Stigma-SCANR was designed as a cross-outbreak stigma mapping tool. This study reports the findings from one of three sites where the Stigma-SCANR was co-developed and implemented with affected community members. The other two sites are communities affected by Ebola disease in Uganda and Nipah virus disease in Bangladesh. In all three contexts, the Stigma-SCANR was developed and administered alongside a set of shorter-form stigma scales (analysed and reported separately [21]).

**Fig. 1:**
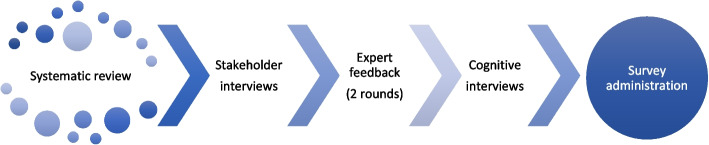
Overview of Stigma-SCANR development process

The development process started with a systematic review of existing outbreak stigma assessment tools [22] and qualitative interviews with 34 stakeholders across a range of acute infectious disease outbreaks [23]. We used the results of these initial steps to develop a draft set of questions. These questions were refined through two iterative rounds of feedback from 41 experts in stigma and re-emerging infectious diseases (Fig. 1). Experts involved in these feedback rounds were invited to score the relevance of each of the final modules to assess content validity, using the content validity index (CVI) scoring system [24]. Scores greater than 0.9 were considered excellent [24]. The overall survey CVI was 0.99 for relevance (average across all modules) and 1.00 for comprehensiveness. Module scores and process details are provided in the supplementary material (Additional file 2: Fig. S1, Tables S2, S3).

The UK mpox specific version of the survey was adapted for the local context and further content validated through 15 pilot cognitive interviews with community members representative of the intended sample (including one community member with a previous diagnosis of mpox) to ensure relevance and understandability. The survey included two attention checks to ensure only valid responses were analysed. This included a standalone instruction disguised as a question, asking respondents to select ‘All’ as their answer to show they were paying attention, and a similar instruction embedded within a matrix of response items. Respondents who failed either attention check were excluded from analyses. Data were collected and stored using Research Electronic Data Capture (REDCap).

#### Content

The UK mpox-specific Stigma-SCANR is a modular 15-minute survey that maps the causes, manifestations, and impacts of mpox stigma (Additional file 3).

The survey primarily focused on anticipated community stigma (how much respondents expect someone with mpox to be stigmatised in their community), as this was deemed most relevant for informing future outbreak control and health policy. This third-person phrasing was used wherever possible, rather than focusing exclusively on the disclosure of personal experiences or attitudes, to minimise participant burden or distress, reduce social desirability bias, and allow reflection on community-level stigma. This approach also enabled inclusion of a broader sampling frame.

Those who reported a previous diagnosis of mpox were directed to an additional module which asked about personal stigma experiences. Stigma was categorised based on the type of manifestation, actor, and target.

In this study, the term affected community is used to refer to a group of individuals with shared context who have direct or indirect experience of the same outbreak [25]. This includes those diagnosed with the illness, their family members and friends, healthcare workers who care for those with the illness, and members of the public in affected areas who identify with or feel connected to the impacted population.

### Ethical considerations

The University of Oxford’s Medical Sciences Division Ethics committee approved this study (reference: R87722/RE004). Data collection was anonymous, and respondents provided informed electronic consent prior to starting the survey. The details of local psychosocial support groups and webpages were provided to participants.

### Patient and public involvement

A community co-investigator, and co-author, with lived experience of mpox was involved from conceptualisation of the study. In addition, 15 community members were involved in reviewing the survey through cognitive interviews. Several community members and organisations assisted with recruitment.

### Data analysis

#### Quantitative data analysis

Descriptive statistics were computed using R software (version 4.3.1). Categorical variables were reported as frequencies and percentages. Age was assessed for normality using visual inspection (histogram, Q–Q plot) and the Shapiro–Wilk test and was summarised using the median and interquartile range (IQR) because it was not normally distributed (*W* = 0.92, *p* < 0.001). REDCap’s required field feature prevented missing data from completed surveys. The quantitative findings were triangulated with qualitative findings. All survey questions analysed are available in Additional file 3.

#### Qualitative data analysis

A mixed deductive and inductive framework analysis was performed on open-ended survey responses using NVivo (release 1.7.2, Lumivero, USA) as a structured approach to managing a large volume of brief textual data [26]. Deductive codes were initially applied based on the existing framework used for the quantitative survey questions, while inductive coding captured any unanticipated emerging themes. The codes were agreed upon by the research team, applied by a team member with qualitative research experience (AP) and the outputs reviewed by all authors. Further supporting quotes for each results component are available in Additional file 4: Table S4.

## Results

### Respondent characteristics

A total of 479 respondents started the survey, with 453 (95%) completing it. After excluding 14 respondents who failed attention checks and two ineligible respondents (one did not live in the UK and the other was not aware of mpox), the final cohort includes 437 respondents (characteristics in Table 1).

**Table 1:** Respondent characteristics

Category	Count (*N* = 437)	Percentage
Age		
Median (IQR, range)	32 (26–41; 18–78)	
Mpox proximity^a^		
Personal lived experience of mpox	13	3
Close relationship with someone who had mpox	42	9
Healthcare worker involved in mpox outbreak response	24	6
Healthcare worker not directly involved in mpox outbreak response	101	23
Other outbreak response worker/support staff	23	5
Community member without the above experiences	271	62
Nature of residence		
Urban	288	66
Rural	149	34
Sex assigned at birth		
Male	306	70
Female	126	29
Other	0	0
Prefer not to say	5	1
Gender identity		
Man	299	68
Woman	86	20
Non-binary	45	10
Other	5	1
Prefer not to say	2	1
Sexual orientation		
Straight	153	35
Bisexual	121	28
Gay	116	27
Other	41	9
Prefer not to say	6	1
Self-reported understanding of mpox		
Heard of it but don’t know details	165	38
Know the basics (e.g. typical symptoms)	223	51
Know more than the basics (e.g. epidemiology)	49	11

*IQR* interquartile range, *N* total number of respondents

^a^Respondents may be included in more than one category

### Stigma characteristics

#### Anticipated stigma

**Fig. 2:**
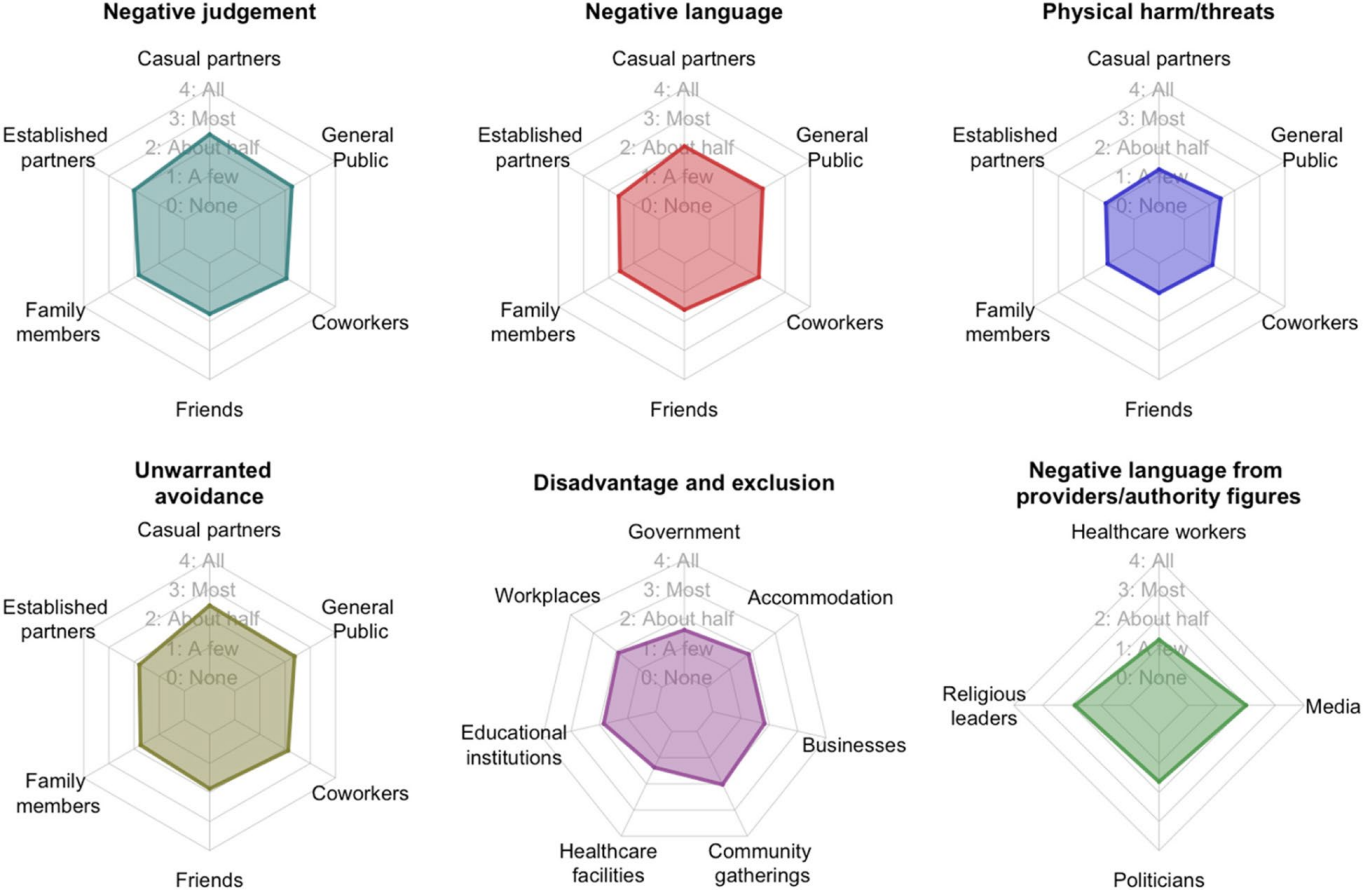
Anticipated stigma by actor and type. Respondents were given a hypothetical scenario where it is known that a person within their community has been diagnosed with mpox. They were then asked to estimate what proportion of the community would enact various forms of stigma. Radar charts show mean scores on the response scale of none (0) to all (4) for different actors. A larger plot is interpreted as more anticipated stigma

Respondents generally expected mpox stigma to be enacted by a minority to half of individuals across social groups (Fig. 2). Negative judgement and avoidance, especially from casual partners and the public, were the most prominent forms of anticipated social stigma (anticipated, on average, from half to most of these individuals) (Fig. 2). Three hundred twenty-one respondents (74%) believed that people within their community would physically harm or threaten someone with mpox.

Similarly, respondents anticipated that, on average, a few to half of all key institutions, including workplaces and healthcare facilities, would disadvantage or exclude someone with mpox (Fig. 2). Almost half of the respondents (195, 45%) believed that if a hairdresser had recently recovered from mpox, few to no customers would get their hair cut by them.

While most respondents (294, 67%) believed the public opinion of someone would return to normal weeks to months after recovery, 15 respondents (3%) felt it would never return to normal.

#### Stigma experiences

Among the 13 respondents who had recovered from mpox, 11 (85%) reported two or more experiences of stigma related to their diagnosis. The most common sources were the public and co-workers or classmates (54% each). Additionally, eight (*n* = 8, 62%) respondents reported feeling stigmatised for a reason other than mpox in the past year, and the same proportion reported that someone close to them had been treated unkindly due to their diagnosis. All but one respondent (*n* = 12, 92%) reported experiencing self-stigma. Open-ended responses included examples of stigma enacted by healthcare providers, mostly involving generalist staff not working in the fields of sexual health or infectious diseases.

### Stigma drivers

#### Anticipated beliefs and emotions

**Fig. 3:**
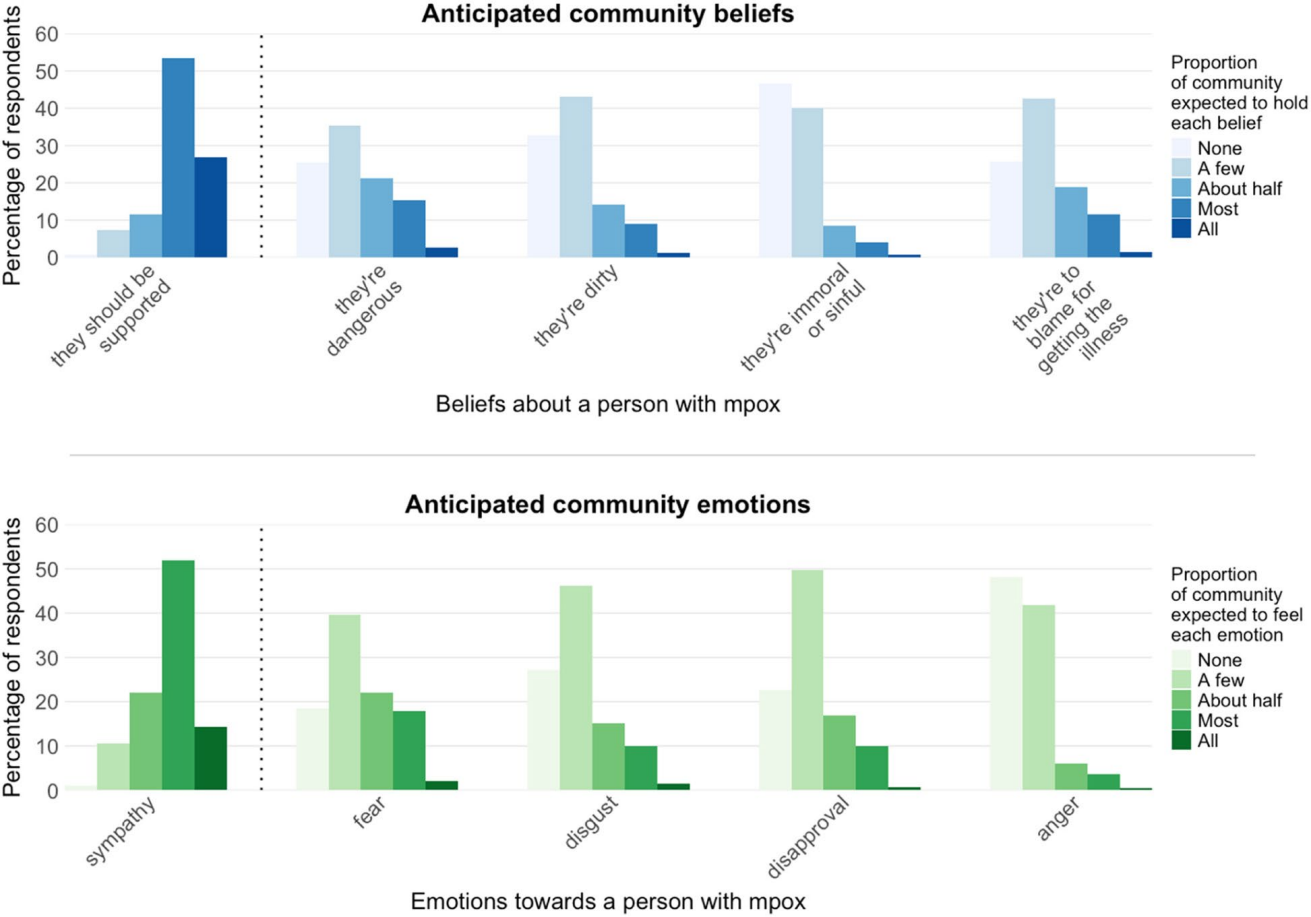
Anticipated community beliefs and emotions towards someone with mpox. Respondents were given a hypothetical scenario where it is known that a person within their community has been diagnosed with mpox (same scenario as Fig. 2). They were then asked to estimate what proportion of the community would hold various beliefs or feelings about the person with mpox

Three hundred fifty-one (80%) respondents believed that the majority (most or all) of their community would be supportive towards someone with mpox, and 290 (66%) anticipated that the majority would feel sympathy (Fig. 3). Among negative beliefs, 79 (18%) respondents expected that most or all people in their community would believe individuals with mpox are dangerous. Other stigmatising beliefs—such as that people with mpox are dirty, immoral, or to blame for getting the illness—were less commonly anticipated as majority views. Similarly, fear was the most frequently anticipated negative emotion (87 respondents, 20%), while fewer respondents expected widespread feelings of disgust, disapproval, or anger (Fig. 3).

These findings were supported in the open-ended questions, with respondents suggesting that the fear related to mpox was primarily due to limited understanding of the disease.

*During [the peak of the outbreak], I saw a lot of homophobia (for example saying gay men were irresponsible or dirty for allowing this outbreak to happen), and anger towards people who were having lots of sex. Most of all, though, I saw fear. I think the anger and hate mostly came from people who were scared of the outbreak.*—Respondent 326, outbreak responder with close relationship to someone with mpox, age 26, North of England

#### Associative stigma

**Fig. 4:**
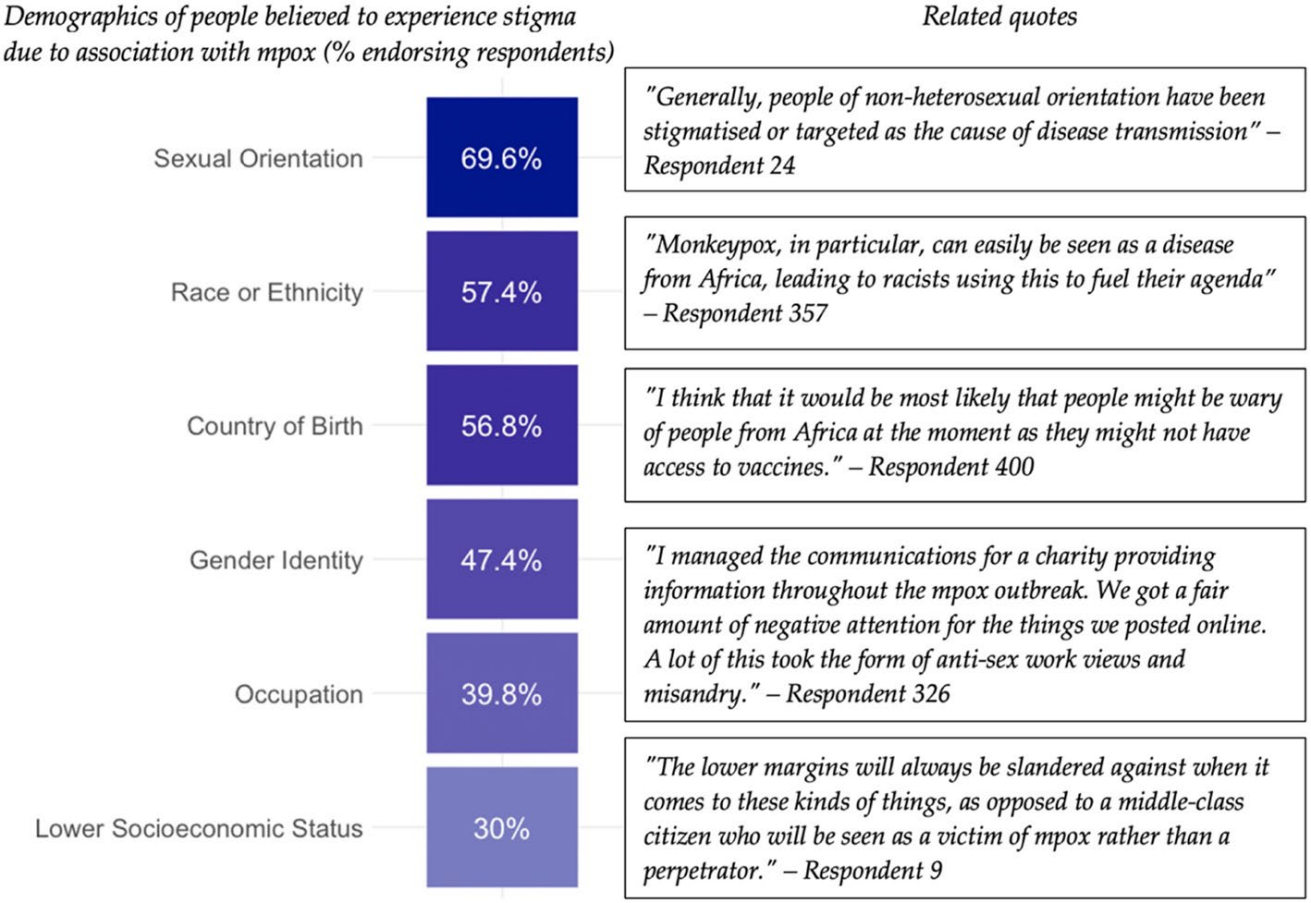
Associative mpox stigma. Respondents were asked if they believed any groups were treated more negatively due to the mpox outbreak. Percentages show proportion of respondents who ticked each option. Related quotes are from an open-text follow-up question asking why the respondents’ ticked the above options

Several minority groups were perceived to face stigma associated with mpox, regardless of whether they personally had mpox (Fig. 4), including certain sexual orientations (304, 70%), people of certain races or ethnicities (251, 57%), and migrants (248, 57%).

Another common theme was the historical and geographical link to other infectious diseases, especially associated with the same population groups:

*It seemed very similar to HIV stigma within the UK in the 90s, and it feels a bit like [we] have not learned anything since.*—Respondent 332, healthcare worker involved in the mpox outbreak response, age 38, South of England

*Everything starts from illness from Africa and spread to Europe, it always sounds as though it’s their fault and we are innocent.*—Respondent 357, priority community member without personal proximity to mpox, age 20, South of England

Respondents felt that aspects of the outbreak response, such as public health messaging, testing criteria, and the illness’s former name, contributed to associative stigma.

*There’s a stigma in the term itself – monkeypox is named after a monkey, which in previous society was a racist term. Even though it may not be consciously in a person’s mind, ethnic minorities might experience stigma because of being associated with an animal, and therefore be seen as unhygienic [or] unintelligent.*—Respondent 82, priority community member without personal proximity to mpox, age 25, Midlands England

## Stigma impacts

### Care-seeking

**Fig. 5:**
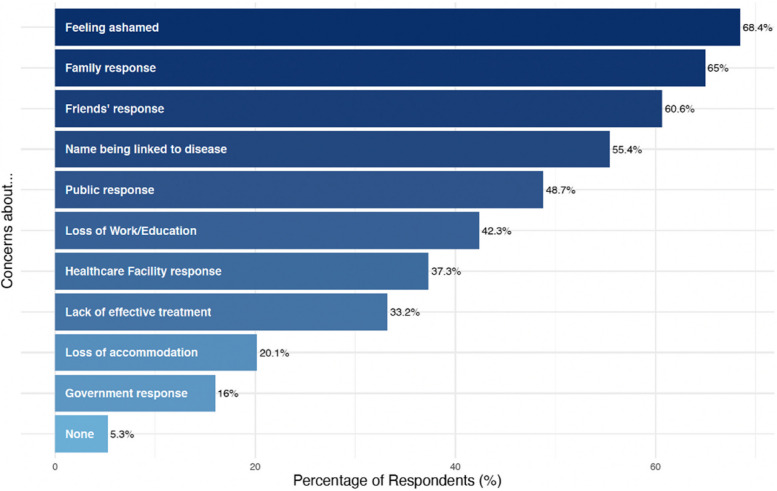
Perceived reasons for avoiding testing for mpox symptoms

Of the 13 respondents who reported a history of mpox, nine (69%) reported seeking testing and/or medical care. Additionally, 23 respondents (6%) reported symptoms they thought may be due to mpox, 16 (70%) of whom sought medical care. Respondents reported a range of stigma-related reasons someone might avoid testing (Fig. 5), the most common being shame (299, 68%).

### Sharing one’s diagnosis

**Fig. 6:**
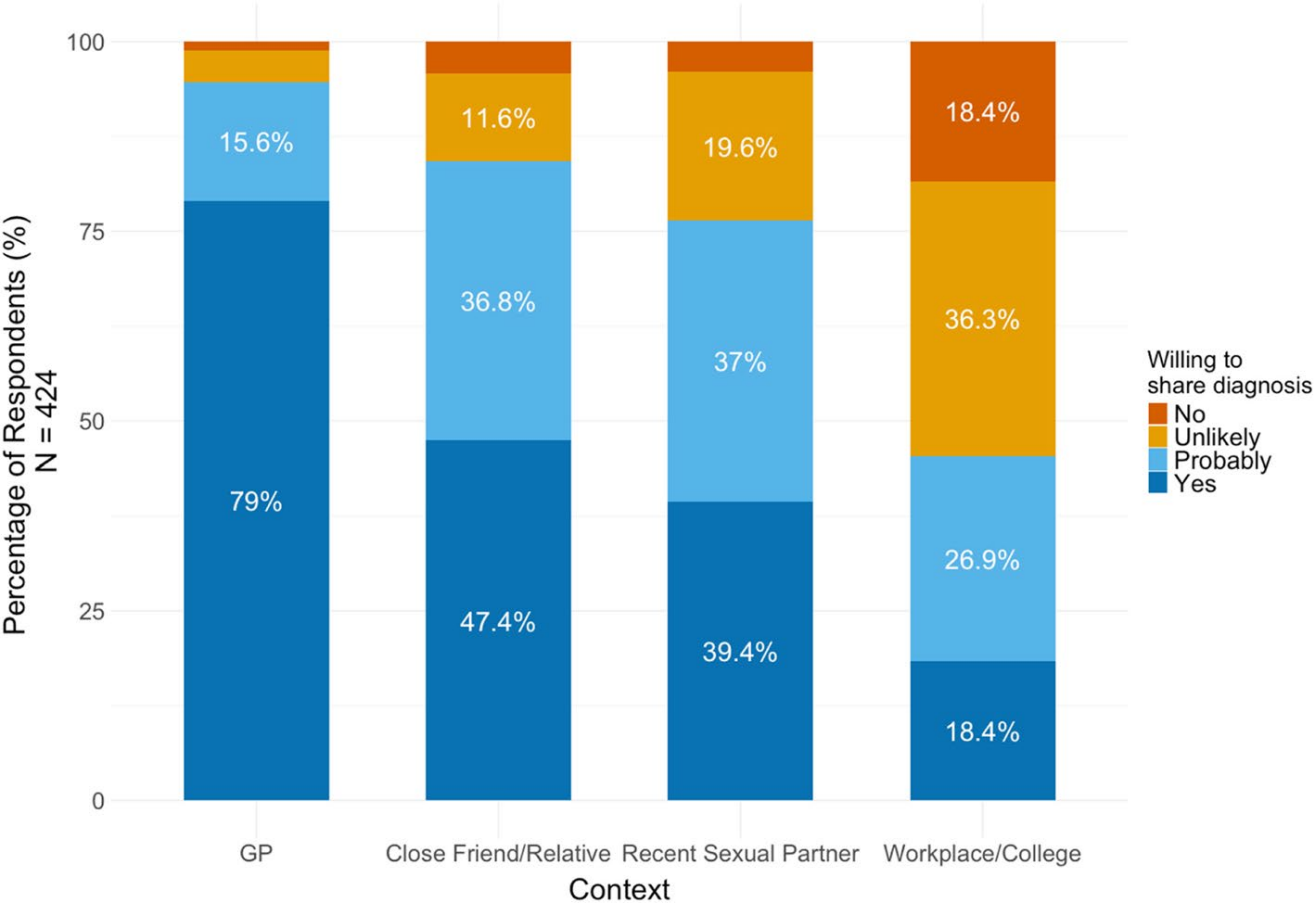
Anticipated willingness to tell others about an mpox diagnosis

Respondents’ anticipated willingness to share a diagnosis of mpox with others varied (Fig. 6). Notably, nearly a quarter of respondents (24%) said they would not or were unlikely to tell a recent sexual partner about an mpox diagnosis.

### Impact on outbreak response worker morale

Of the 31 healthcare workers and support staff directly involved in responding to the mpox outbreak, 6 (19%) reported feeling negatively judged or treated unkindly due to their involvement in the outbreak response, with another 5 (16%) selecting ‘prefer not to say’. Two outbreak responders (7%) said they considered changing occupations because of how they were treated during their involvement in the mpox response.

## Stigma reduction avenues

**Fig. 7:**
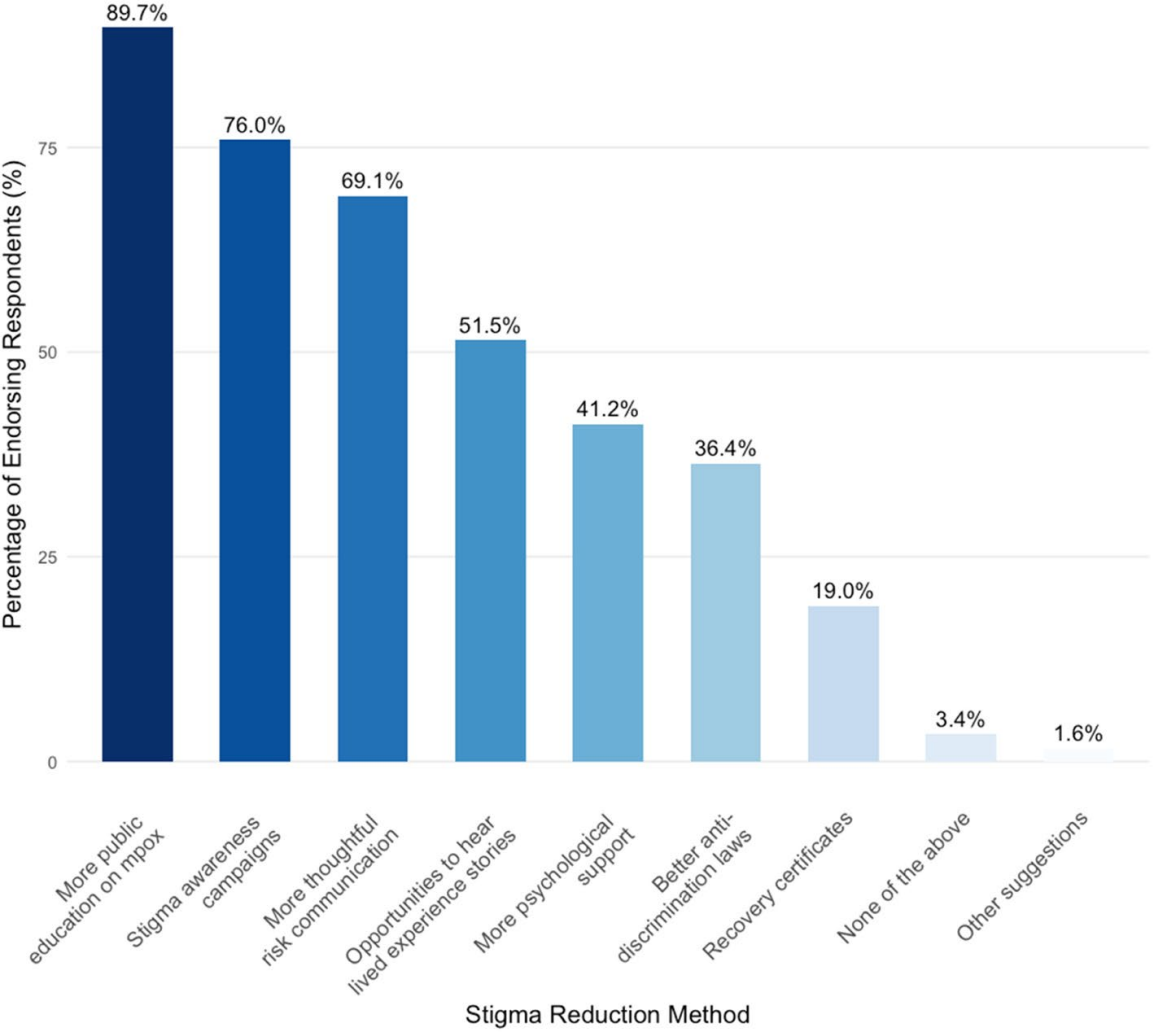
Endorsement of stigma reduction strategies. Respondents were asked which measures they believe would be helpful to further reduce stigma. Respondents were able to select all that applied and provide alternative open-text suggestions

The most supported future stigma reduction intervention was more public education (392 respondents, 90%), followed by stigma awareness campaigns (332 respondents, 76%) and then more thoughtful risk communication (302 respondents, 69%) (Fig. 7).

Several respondents identified a link between stigma and the lack of education about mpox, suggesting that this allows for the spread of misinformation:

*I genuinely believe that the stigma attached is generally due to uneducated people unfortunately spreading misinformation.*—Respondent 348, healthcare worker with close relationship with someone with mpox, age 31, South of England

*As a worker in a hospital my fear is always how cases like this are moved around [and] come in contact with the wrong staff who are not informed*.—Respondent 239, healthcare worker not directly involved in mpox outbreak response, age 37, South of England

These responses emphasised the need for targeted education, both for the general public and for hospital staff not directly involved in GBMSM care or outbreak response.

Respondents emphasised that although mainstream and social media was a potential driver of stigma it could similarly play an important role in reducing stigma. One respondent gave an example of how social media and dating applications can also be an accessible avenue for stigma reduction.

*The only support [for those with] monkeypox I’ve seen was on Grindr*.—Respondent 249, priority community member without personal proximity to mpox, age 22, South of England

Most respondents (7, 54%) with a previous mpox diagnosis reported wanting to speak to someone else who had experienced the illness, while four (31%) expressed a motivation to help others who may have the illness. Six respondents (46%) said that having mpox made them willing to speak about their experience publicly.

## Discussion

This study adds to the emerging literature on mpox by offering insights into how and why stigma became a pervasive feature of the outbreak in the UK, and how this shaped both personal experiences and outbreak dynamics. Consistent with previous survey data [4], our findings identify substantial anticipated social and structural stigma among affected communities, including expectations of negative judgement, avoidance, and exclusion in both community and institutional contexts. The results build on this literature, suggesting that stigma was largely driven by a combination of fear, misinformation, and entrenched biases. The findings also highlight the impact of stigma on both individual well-being and outbreak control, particularly the role of shame in deterring care-seeking.

Respondents’ consistent emphasis on associative stigma towards minority groups underscores why mpox-related stigma must be understood within broader patterns of marginalisation and the continuing risk of intersectional harm. The risk of intersectional stigma for LGBTQ + groups has been highlighted since the start of the outbreak and is both reminiscent of, and compounded by, HIV-related stigma [27, 28]. Additionally, the potential for the mpox outbreak to be exploited for political purposes and anti-LGBTQ + agendas has been emphasised [27, 28], a concern echoed in qualitative responses to our survey. This issue is increasingly relevant as new cases emerge in countries with anti-LGBTQ + laws or who face high rates of violence against this population [27, 29, 30].

Our study results also point to other drivers of mpox stigma. Interestingly, while existing literature emphasises perceptions of dirtiness or immorality as key beliefs driving mpox stigma [10, 11, 29], our findings suggest that perceptions of danger, and associated fear, were more prevalent. This indicates that addressing fear through clear public communication may be an effective strategy for reducing stigma. This approach also ensures risk communication reaches populations who may be at higher risk but who do not engage with LGBTQ + resources or sexual health clinics, including discrete MSM [4, 9].

A concerning finding was that anticipated mpox stigma frequently included concerns not only about individual attitudes, but also related to institutional and socioeconomic barriers. Although self-isolation periods have been shortened since the outbreak began [9], two out of five respondents still viewed concerns about work or education loss as barriers to seeking care. While anti-stigma recommendations are often limited to advice on language use, these findings underscore the need for more comprehensive strategies to address structural discrimination and prevent deepening health and economic inequalities [27]. They also highlight the importance of collecting data on structural discrimination, which is often missing from stigma measures [11, 29].

### Implications for future outbreak control

Table 2 outlines potential implications of survey findings, considered alongside existing literature, for informing future outbreak control efforts.

**Table 2:** Suggestions for future mpox outbreak response based on stigma survey findings and existing literature

Survey finding	Relevant existing literature	Suggestions for future outbreak control
*Underlying causes and facilitators*		
Fear was the most anticipated negative emotional response to someone with mpox. Mpox stigma was most frequently anticipated from members of the public	Logie [31] describes how mpox stigma is linked to three archetypes: the ‘foreign’ other, the ‘immoral’ other, and the ‘unwell’. The first two archetypes require deeper system change, but stigma towards the ‘unwell’ is often rooted in fear, which can be mitigated through clear information on risk and prevention	• While it is important for risk communication and prevention programmes to be focused on populations most affected, ensuring the broader public understand the modes of transmission and preventive measures may also help alleviate fear-driven stigma • Information emphasising the importance of community support could be incorporated into public health programmes • Including people with lived experience in public response from early in an outbreak could help humanise the outbreak and reduce fear
Misinformation and harmful portrayals of mpox by a vocal minority of traditional and social media were thought to contribute to mpox stigma	Social media posts that communicated erroneous information on mpox began earlier and grew faster in the outbreak, reaching a wider audience than later communications by health officials [32]. Community notes added to posts increase trust in fact-checking on social media [33]. In the UK, the most trusted sources of information on mpox were healthcare professionals, official health agencies, and mainstream media [28]	• Encouraging community-based fact-checking could help counter misinformation • Early dissemination of best-available information by health institutions, healthcare providers, and trusted media outlets is important for counteracting the rapid rise in misinformation • Images linked to mpox communications should represent the diversity of affected population groups
Pre-existing prejudices against GBMSM and migrants were found to be important drivers of stigma. Public health communications that link key populations to mpox without context were thought to exacerbate stigma	In a qualitative study with populations most affected [9], there was a unanimous suggestion that risk communication should be broadly inclusive and disentangle higher-risk behaviours from sexual identity	• Messaging that describes ‘higher risk’ populations should aim to clearly explain why (e.g. current transmission patterns) • Risk communication should aim to clarify behaviours that are associated with increased risk of transmission within relevant networks rather than centring on demographic labels • Public health programmes should be evidence-based and collaboratively developed with the population groups being referenced
*Manifestations*		
Nearly all respondents with a previous mpox diagnosis reported experiencing self-stigma. Over half of recovered respondents reported wanting to speak to someone else who had experienced the illness, while a third expressed a motivation to help others affected. Feelings of shame were considered the most common barrier to care-seeking	Feelings of guilt and shame have been frequently reported by those affected by mpox [4]. Witzel et al. [10] found that older men who were living with HIV were able to draw on resilience from experience of HIV diagnosis to resist internalising the stigma they experienced	• Psychological support should be available to mpox patients, with information on available support accessible to those hesitant to seek care, such as via dating apps • Funding and facilitating peer support and community involvement may be particularly valuable, though these should consider the increased identifiability and discrimination risks for those involved • Individuals with experience in HIV advocacy and peer support may offer valuable, transferable skills to mpox outbreak response efforts
Mpox was expected to have a negative socioeconomic impact on many affected, including following recovery. For example, nearly half of respondents believed that few to no customers would visit a hairdresser who had recently recovered from mpox	Reports have documented instances of individuals losing their jobs and being ‘outed’ as GBMSM due to extended isolation periods [4]	• Access to social support services may help mitigate longer-term impacts of stigma among affected individuals • Providing clear information on recovery timelines and safe return to work or school could help reduce uncertainty and support reintegration • Legislative measures to prevent discrimination in work and educational settings, particularly around medical leave for isolation, may help protect those recovering from mpox
There were concerns and reports of stigma enacted by a small subset of healthcare workers, primarily those not usually involved in sexual health services or outbreak response	People with mpox have previously reported stigmatising attitudes from healthcare professionals who lacked skills in supporting GBMSM [4, 10]. Positive experiences of healthcare for GBMSM have also been described as limited to designated sexual health settings [34]	• Establishing or adapting systems to ensure clear avenues for patients to report stigmatising experiences may help address stigma in healthcare settings. This can be done in the absence of an outbreak and need not be disease specific • Ensuring all healthcare and support staff who may come into contact with people with the illness have access to accurate, up-to-date outbreak information may support more inclusive and informed care
Nearly three quarters of respondents anticipated that someone with mpox would be physically harmed or threatened by at least a few people in their community. Disgust, disapproval, and anger were also anticipated to be minority emotional responses to mpox	Existing stigma surveys typically only capture majority views, with questions such as ‘Most people believe that a person with mpox is dirty’ and an agreement scale [28, 35]	• Stigma assessment tools used to inform risk communication and community engagement should effectively distinguish between majority and minority attitudes and behaviours • Minority sentiments may still have a substantial impact and should be considered in the design of stigma monitoring and response efforts
*Impacts on outbreak control*		
Nearly a quarter of respondents said they would not or were unlikely to share their diagnosis with a recent sexual partner	A case report of the first mpox case in Northeast Italy describes the reluctance to engage with contact tracing teams due to disclosure concerns [36]	• Contact tracing efforts could benefit from co-design with affected communities and offering and emphasising anonymity
Nearly 20% of healthcare workers involved in the outbreak reported feeling negatively judged or mistreated. Two outbreak responders said they considered changing occupations due to how they were treated	In an earlier survey of UK sexual health professionals [37], 35.8% reported that their experience during the 2022 mpox outbreak made them less likely to stay in their profession. Reasons included understaffing and frustration with the outbreak response [37]	• Providing additional support and resources to sexual health services and professionals is indicated during outbreaks with sex-related modes of transmission

### Strengths and limitations

This study offers valuable insights into mpox stigma in the UK but should be interpreted in light of its methodological limitations. This study used purposive sampling to represent groups most affected by the mpox outbreak. While this approach provided specific insights, and likely contributed to the high response rate, it limits generalisability due to non-random sampling. To mitigate the impact of potential response bias, questions were designed to explore community-level perceptions, indirectly capturing the views of individuals less likely to participate in stigma-related surveys.

A key strength is the adaptability of the Stigma-SCANR survey design, enabling rapid deployment in future outbreaks. This is particularly important for mpox, given the diversity of virus clades, modes of transmission, at-risk groups, and settings where the disease occurs. Nonetheless, the findings are specific to the UK and may differ in regions with varying mpox clades, cultural contexts, and affected populations.

Small sample sizes restricted comparative analyses, and the sample of individuals with mpox was particularly small. The findings therefore primarily relate to anticipated stigma among directly and indirectly affected communities. The lack of sub-analyses also meant we could not assess differences in response patterns between healthcare workers and, for example, respondents who identify as LGBTQ + . Lastly, recall bias may have influenced responses due to the time elapsed since the peak of the mpox outbreak.

### Future research implications

Further research is needed to explore how mpox stigma manifests in diverse settings and to evaluate interventions that can reduce its impact over time. Future research is specifically needed on mpox stigma in regions experiencing sustained or recent transmission, where the affected community profile differs from the UK. Longitudinal studies could help track the long-term impacts of mpox stigma on mental health, employment, and social reintegration. Co-design of studies and outbreak response efforts with affected community members is important for respecting the priorities of those affected and for improving acceptance and uptake. The feasibility and value of co-designing outbreak response measures are illustrated by the successful co-creation of a mpox communication campaign in the WHO European Region in partnership with key affected communities [38]. Rigorous evaluation of stigma reduction interventions and outbreak response adaptations are required to ensure efforts to reduce stigma are evidence-based.

## Conclusion

Together, these findings add to the existing understanding of mpox stigma and highlight the importance of addressing both anticipated and experienced stigma in future public health responses. Reducing disease-related stigma is an attainable and important means of improving the well-being and safety of people affected by mpox, and simultaneously enhancing outbreak response. This is crucial for ongoing global outbreak control efforts and any future resurgences of mpox.

## Supplementary Information

Additional file 1: Table S1 Checklist for Reporting Of Survey Studies (CROSS).

Additional file 2: Details of expert feedback for content validation of survey. Fig. S1 Expert iterative feedback process. Table S2 Content validation expert characteristics. Table S3 Final content validity scores.

Additional file 3: Stigma-SCANR (UK mpox specific version).

Additional file 4: Table S4 Additional qualitative quotes.

## Data Availability

The datasets used and/or analysed during the current study are available from the corresponding author on reasonable request.

## Abbreviations

CROSS: Consensus-Based Checklist for Reporting of Survey Studies
GBMSM: Gay, bisexual, and other men who have sex with men
IQR: Interquartile range
MPXV: Mpox virus
PHEIC: Public Health Emergencies of International Concern
REDCap: Research Electronic Data Capture
Stigma-SCANR: Stigma Survey and Community-based Assessment for New and Re-emerging outbreaks
UK: United Kingdom
WHO: World Health Organization
