# Supplementary material for: Mpox stigma in the UK and implications for future outbreak control: a cross-sectional mixed methods study
Source: BMC Med. 2025 Jul 15;23:422. doi: 10.1186/s12916-025-04243-3 (PMC12261657; doi:10.1186/s12916-025-04243-3)
Supplement: Supplementary file 2 — Additional file 2: Details of expert feedback for content validation of survey. Fig. S1 Expert iterative feedback process. Table S2 Content validation expert characteristics. Table S3 Final content validity scores. [file 12916_2025_4243_MOESM2_ESM.pdf]

Additional File 2: Details of expert feedback for content validation of survey
Figure S1: Expert iterative feedback process

Round 1
Experts sent initial survey with questions adapted from literature and stakeholder interviews.

Experts asked to rate:
- Clarity (C) of each question: scale of 1-4 (if <3, asked for suggested rephrasing)
- Relevance (R) of each question: 1-4 (if <3, asked for reason)
- Comprehensiveness: 1-4 (if <4, asked about missing components)

Also asked for feedback on:
- Instructions and response options
- Modular structure
- Length of survey

➢ Content Validity Index (CVI) calculated for each question:
- If >80% for clarity and <80% for relevance: question excluded (0/11 questions)
- If >80% for relevance and <80% for clarity: major revisions and included in Round 2 (6/11 questions)
- If >80% for both: included with minor revisions based on consistent feedback (5/11 questions)
- If <80% for both: major revisions when possible, otherwise excluded (0/11 questions)
- Additional suggested questions added (41% experts thought all components were assessed in initial survey)

Round 2:
Experts sent revised survey (with controlled feedback (e.g., 86% scored this item quite or highly relevant).
Experts asked to score questions and provide feedback as in Round 1.

➢ CVI calculated and questions revised as per Round 1 criteria
- All questions scored >80% for clarity and relevance
- 95% experts thought all components were assessed

Round 3
Experts sent final form survey for content validation after translation, community review (including clarity assessment by intended respondents), field-testing, and psychometric analysis
Experts asked to rate:
- Relevance of each question/module: 1-4 (if <3, asked for reason)
- Comprehensiveness: 1-4 (if <4, asked about missing components)
- Also asked for feedback on Instructions, response options, face validity and comprehensiveness

➢ Calculate question and survey relevance CVI & adjust with modified Kappa
➢ Calculate survey comprehensiveness CVI
➢ If any <80% identify cause of concern from open text feedback

CVI = Content validity index

Table S2: Content validation expert characteristics

Expert characteristics	Number of experts		
	Round 1 (N = 41)	Round 2 (N = 39)	Round 3 (N = 35)
**WHO region***			
African Region	9	9	8
Region of the Americas	6	5	5
Eastern Mediterranean Region	2	2	2
European Region	10	9	6
South-East Asia Region	8	8	7
Western Pacific Region	6	6	6
**Outbreak response experience (disease$)**			
Mpox and other orthopoxviruses	12	10	8
Viral haemorrhagic fevers (e.g., Ebola))	40	38	33
COVID-19	12	11	11
Other novel coronaviruses (SARS, MERS)	5	5	5
Nipah virus and other henipaviruses	4	4	4
Zika virus	5	5	5
Influenzae (all strains)	6	6	6
Cholera	5	5	5
Dengue	7	7	7
Plague	1	1	1
Hepatitis (A or E)	2	2	2
Chikungunya	1	1	0
**Outbreak response experience (location)**			
Local (in-country)	35	34	30
Regional/Global	10	9	6
**Role in outbreak**			
Psychosocial response team	11	11	10
Clinical response team	12	11	10
Social science research	25	25	21
Clinical research	11	10	8
Risk communication and community engagement	20	18	16
Patient advocacy	6	5	4
National Policy (e.g. Ministry of health)	9	9	8
International Policy and Advocacy	8	8	6

**coded by nationality, 10 experts from each region invited to provide feedback, $original list based on WHO R&D blueprint with option to specify 'other', experts could select more than one option for all categories*

Table S3: Final content validity scores

Module Number	Module content	Expert relevance CVI score	Interpretation
1	Care-seeking and stigma	1.00	Excellent
2	Beliefs and feelings	1.00	Excellent
3	Anticipated social stigma	1.00	Excellent
4	Anticipated structural stigma	1.00	Excellent
5	Personal experiences of stigma	1.00	Excellent
6	Concerns about sharing diagnosis and social acceptance	0.97	Excellent
7	Stigma by association	1.00	Excellent
8	Stigma reduction	0.97	Excellent
9	Stigma narratives	0.97	Excellent

CVI = content validity index; interpretation: >0.8 considered adequate; >0.9 considered excellent

Overall average survey-content validity index (s-CVI): 0.97

Overall survey comprehensiveness score: 1.00
