# Supplementary material for: Mpox stigma in the UK and implications for future outbreak control: a cross-sectional mixed methods study
Source: BMC Med. 2025 Jul 15;23:422. doi: 10.1186/s12916-025-04243-3 (PMC12261657; doi:10.1186/s12916-025-04243-3)
Supplement: Supplementary file 3 — Additional file 3: Stigma-SCANR (UK mpox specific version). [file 12916_2025_4243_MOESM3_ESM.pdf]

Additional File 3: Stigma-SCANR (UK mpox specific version)

Branching logic indicated by symbols (e.g., *,#)

Thank you for your interest in this survey on **mpox stigma**. This survey is run by researchers from the University of Oxford.

Mpox stigma happens when people look down on someone or treat them unkindly because they associate them with mpox (previously known as monkeypox).

Some important details before you begin:

1. We would like people to answer this survey if they are 18 years or older and have heard about mpox. **You don't need to have had personal experience of mpox** to take part in the survey.
2. It is voluntary to do the survey.
3. It usually takes about **15 minutes** to complete.
4. Your responses will be kept anonymous. We will not ask for your name or contact details. Your IP address will not be stored.
5. You can come back to the same survey as many times as you need before submitting.
6. Although it will not immediately benefit you, taking part in the survey will help us to reduce stigma and assist people who feel stigmatised in the future.
7. There is a risk that some of the questions remind you of difficult personal experiences. If this is the case for you, you can take a break or stop the survey. You can access resources for psychological or social support *here*.
8. Submitting the survey will be taken as permission to store and use your anonymous responses for research. This research will be published in an open-access journal and form part of a PhD.
9. Please only take the survey once.
10. This research has received ethics clearance from the University of Oxford Medical Sciences Division ethics committee (reference number: R87722/RE001).

If you have any questions or concerns about this survey please speak to Amy Paterson (**amy.paterson@ndm.ox.ac.uk**) or Piero Olliaro (**piero.olliaro@ndm.ox.ac.uk**). We will acknowledge your question or concern within 10 working days. You can also contact the Chair of the Research Ethics Committee at ethics@medsci.ox.ac.uk

Are you happy to proceed? Yes/No

Background details

- Age: __ years
- Residential region: Midlands, England/Northern Ireland/North of England/Scotland/South of England/Wales/I do not live in the UK [end survey]
- Nature of residence: Urban (city/large town)/Rural (small town/village)
- Sex assigned at birth: Male/Female/Other/Prefer not to say
- Gender identity: woman/man/non-binary/other: please specify/prefer not to say
- Sexual orientation: straight/gay/lesbian/bisexual/asexual/other: please specify/prefer not to say

- Have you received an mpox vaccine? Yes/No/Unsure/Prefer not to say

- Are you a healthcare worker? Yes*/No#
- #Have you done work related to an infectious disease outbreak? Yes*/No
- *Have you been involved in responding to the mpox outbreak? Yes/No

- How would you rate your understanding of mpox?
- Never heard of it [end survey]
- Heard of it but don’t know many details
- Understand the basics (e.g., how it is passed on and typical symptoms)
- Know more than the basics

- Do you know of anyone close to you who has had mpox? Yes/No/Prefer not to say

Module 1: Care-seeking and stigma

1a. If willing to share, have you ever had mpox? Yes*/No§/Unsure§/Prefer not to say
1b. §Have you ever had symptoms that you thought may be due to mpox? Yes#/No/Prefer not to say
1c. *#Did you seek medical attention? Yes&/No/Prefer not to say
1d. &How long after you had the first symptom did you seek medical attention? XX days/No symptoms when tested/Prefer not to say

1e. In your opinion:
Which of the following are reasons someone with mpox symptoms may avoid seeking medical attention? (select all that apply)

☐ how their family members might respond
☐ how friends might respond

- ☐ how the public might respond
- ☐ how the government might respond
- ☐ concerns about losing accommodation
- ☐ concerns about losing work or education opportunities
- ☐ concerns about unkind treatment at the healthcare facility
- ☐ concerns about their name being linked to the disease
- ☐ concerns about feeling ashamed
- ☐ concerns that nothing can be done to help
- ☐ none of the above

1f. If you had symptoms of mpox today, would you choose to delay or avoid seeking medical attention for any of the above reasons? Yes/No/Unsure/Prefer not to say

Module 2: Beliefs and feelings about people with mpox

Note: For the purposes of this survey 'your community' means the people you regularly interact with.

Imagine someone in your community has just been diagnosed with mpox and everyone knows.

In your opinion:
2a. **How many people would believe that the person with mpox…**

		None	A few	About half	Most	All
2a.1.	should be supported	o	o	o	o	o
2a.2.	is to blame for getting mpox	o	o	o	o	o
2a.3.	is immoral or sinful	o	o	o	o	o
2a.4.	is dirty	o	o	o	o	o
2a.5.	is cursed	o	o	o	o	o
2a.6.	is dangerous	o	o	o	o	o

2b. How many people would feel…

		None	A few	About half	Most	All
2b.1.	Sympathy for the person with mpox	o	o	o	o	o
2b.2.	Disapproving of the person (look down on them)	o	o	o	o	o
2b.3.	Afraid of the person	o	o	o	o	o
2b.4.	Disgusted by the person	o	o	o	o	o
2b.5.	Angry with the person	o	o	o	o	o

Module 3: Anticipated social stigma

In your opinion:

3a. How many of the following people might **negatively judge** a person because they have mpox?

		None	A few	About half	Most	All
3a.1.	Casual/potential sexual partners	o	o	o	o	o
3a.2.	Established partners/spouses	o	o	o	o	o
3a.3.	The person's family members	o	o	o	o	o
3a.4.	The person's friends	o	o	o	o	o
3a.5.	The person's co-workers/classmates	o	o	o	o	o
3a.6.	General public	o	o	o	o	o

3b. How many of the following people might **talk badly about** the person known to have mpox (including insulting language by message or social media)?

		None	A few	About half	Most	All
3b.1.	Casual/potential sexual partners	o	o	o	o	o
3b.2.	Established partners/spouses	o	o	o	o	o
3b.3.	The person's family members	o	o	o	o	o
3b.4.	The person's friends	o	o	o	o	o
3b.5.	The person's co-workers/classmates	o	o	o	o	o
3b.6.	General public	o	o	o	o	o
3b.7.	**Healthcare workers**[1]	o	o	o	o	o
3b.8.	**Religious/community leaders**	o	o	o	o	o
3b.9.	**Politicians**	o	o	o	o	o
3b.10.	**The media/journalists**	o	o	o	o	o

[1]including those working in clinics, GP practices, ambulances, hospitals, and treatment units

3c. How many of the following people might **physically harm or threaten to harm** the person with mpox or their property (including once recovered)?

		None	A few	About half	Most	All
3c.1.	Casual/potential sexual partners	o	o	o	o	o
3c.2.	Established partners/spouses	o	o	o	o	o
3c.3.	The person's family members	o	o	o	o	o
3c.4.	The person's friends	o	o	o	o	o

3c.5.	The person's co-workers/classmates	o	o	o	o	o
3c.6.	General public	o	o	o	o	o

In your opinion:
3d. How many of the following people might **avoid** a person known to have **recently recovered from mpox** (i.e., cannot pass the condition on)?

		None	A few	About half	Most	All
3d.1.	Casual/potential sexual partners	o	o	o	o	o
3d.2.	Established partners/spouses	o	o	o	o	o
3d.3.	The person's family members	o	o	o	o	o
3d.4.	The person's friends	o	o	o	o	o
3d.5.	The person's co-workers/classmates	o	o	o	o	o
3d.6.	General public	o	o	o	o	o

3e. How many people in the community might tell others about a person's mpox diagnosis without their permission?

None	A few	About half	Most	All

3f. If people in your community heard that a hairdresser had recently recovered from mpox how many might still get their hair cut by them?

None	A few	About half	Most	All

3g. When do you think the public opinion of someone who had mpox would return to the same as before?
o Less than a week after recovery
o Weeks to months after recovery
o More than a year after recovery
o Never

Module 4: Anticipated structural stigma

4. How many of the following places/institutions might **disadvantage or exclude** people known to have **recently recovered from mpox**?

	None	A few	About half	Most	All

4.1.	Government facilities	o	o	o	o	o
4.2.	Workplaces	o	o	o	o	o
4.3.	Schools or colleges	o	o	o	o	o
4.4.	Healthcare facilities (e.g., clinics or hospitals)	o	o	o	o	o
4.5.	Community gatherings/groups	o	o	o	o	o
4.6.	Businesses (e.g., shops/bars/gyms)	o	o	o	o	o
4.7.	Accommodation (e.g., landlords)	o	o	o	o	o

Module 5: Personal experiences of mpox stigma

[branching logic: only shown if yes to personal experience in demographics]

You can choose 'prefer not to say' for any of these questions

5a. How many times have you experienced the following due to association with mpox?

		Never	Only once*	A few (2-5) times*	Many (>5) times*	Prefer not to say*
5a.1.	Negative attitudes towards you	o	o	o	o	o
5a.2.	Insulting or inappropriate language (directly towards you or used to describe you, including on social media)	o	o	o	o	o
5a.3.	Physical harm or threats of harm (to you or your property)	o	o	o	o	o
5a.4.	People avoiding you even when there is no risk of you giving them mpox	o	o	o	o	o
5a.5.	Disadvantage or exclusion	o	o	o	o	o

5b. *If you are willing to share, who acted in the above way(s) towards you? Select all that apply

Family member(s)/Friend(s)/Neighbour(s)/Co-worker(s)/Classmate(s)/Religious or community leader(s)/Politician(s)/General public/Other/Prefer not to say

5c. Select all that are true in your experience (at any point since diagnosis):

Having mpox made me…

- ☐ Feel ashamed
- ☐ Feel less confident
- ☐ Blame myself for getting mpox
- ☐ Avoid speaking to friends or family
- ☐ Avoid social gatherings even when recovered

- ☐ Want to talk to someone who had recovered from mpox
- ☐ Want to help others who may have mpox
- ☐ Willing to share my experience of mpox publicly
- ☐ Prefer not to say

5d. Was anyone close to you (e.g., partner, family) ever treated unkindly because of your mpox diagnosis? Yes/No/Unsure/Prefer not to say

5e. Have you ever felt stigmatised (looked down on or treated badly) because of a reason other than mpox in the past year? Yes/No/Prefer not to say

Module 6: Concerns about sharing diagnosis and social acceptance
[*branching logic: only shown if no personal history of mpox*]**:**

6a. If you had mpox, do you think you would feel comfortable telling the following people about the diagnosis?

		Yes	Probably	Unlikely	No
6a.1.	a GP	o	o	o	o
6a.2.	a recent sexual partner	o	o	o	o
6a.3.	a close friend/family member	o	o	o	o
6a.4.	your workplace/school/college	o	o	o	o

6b. Please select all that apply below[1]:
I would be willing to accept someone who has recently recovered from mpox as…

- ☐ a new close friend
- ☐ my doctor
- ☐ a new neighbour on the same street
- ☐ a tourist in my country

[1] Question adapted from Bogardus Social Distance Scale: Bogardus E. S. (1925. b). Social distance and its origins. Journal of Applied Sociology, 9, 216–226. https://babel.hathitrust.org/cgi/pt?id=inu.30000104215342&view=1up&seq=226&q1=bogardus

Module 7: Stigma by association

7a. Do you think any of the following groups are treated negatively due to the mpox outbreak?

- ☐ People of certain sexual orientations (e.g., gay, lesbian, bisexual)
- ☐ People of certain gender identities (e.g., men, women, nonbinary) or trans people
- ☐ People with certain occupations (e.g., healthcare workers, sex workers)
- ☐ People from certain countries
- ☐ People of certain races or ethnicities
- ☐ People with less money than others in the community
- ☐ Other
- ☐ None of the above

Please briefly specify why you selected the options above: [open text]

[*Branching logic: Next questions only shown if yes to mpox responder question*]

7b. Did you ever feel negatively judged or treated unkindly due to your involvement in responding to the mpox outbreak? Yes/No/Prefer not to say

7c. Did the way you were treated during the mpox outbreak ever make you consider changing occupations? Yes/No/Prefer not to say

Module 8: Stigma reduction

8. In your opinion:

Which of the following would help reduce mpox stigma? (select all that apply)

- ☐ More public education about mpox
- ☐ More thoughtful public health messages
- ☐ A recovery certificate for those affected
- ☐ More psychological support
- ☐ More laws to stop discrimination
- ☐ Opportunities to hear the stories of people who have recovered from mpox
- ☐ Awareness campaigns about mpox stigma
- ☐ Other: please specify

Module 9: Stigma narratives:

9a. Are there any stories you have heard or seen shared about mpox or people with mpox that you think contribute to stigma? If so, please share them here: [open text]

9b. Are there any stories you would like people to share about mpox or people with mpox? If so, please share them here: [open text]
