# Supplementary material for: Mpox stigma in the UK and implications for future outbreak control: a cross-sectional mixed methods study
Source: BMC Med. 2025 Jul 15;23:422. doi: 10.1186/s12916-025-04243-3 (PMC12261657; doi:10.1186/s12916-025-04243-3)
Supplement: Supplementary file 4 — Additional file 4: Table S4 Additional qualitative quotes. [file 12916_2025_4243_MOESM4_ESM.pdf]

Additional File 4
Table S4: Additional qualitative quotes

Component of stigma	Additional qualitative quotes
Drivers	*"People often use any excuse to demonise minorities and I think mpox could be used as one of these excuses"* – Respondent 180, Northern Ireland<br><br>*"The testing criteria for mpox unfortunately exacerbates the stigma"* – Respondent 337, Wales<br><br>*"The media is doing a good job of stirring up fear."* – Respondent 405, Midlands England
Manifestations	*"A friend of mine contracted mpox. After disclosing his diagnosis, he faced significant backlash and ostracism from his community and workplace"* – Respondent 58, Midlands England<br><br>*"I can imagine that minorities may be treated differently by a few people [due to mpox] but I don't believe this would be the norm."* – Respondent 254, Scotland<br><br>*"There was a lot on social media about certain ethnicities having mpox and LGBT people. There were horrible things said."* – Respondent 54, South of England
Impact	*"Mpox has the potential to negatively affect and further isolate already marginalised groups of people."* – Respondent 2, South of England<br><br>*"I think groups that would already be vulnerable to harassment are treated worse if [there is] an outbreak."* – Respondent 158, Midlands, England<br><br>*"My friend had it and people knew and they kept on spreading [the news of his diagnosis] till everyone knew it. It made him have low self esteem"* – Respondent 437, Northern Ireland
